# Peer Support in Mental Health: Literature Review

**DOI:** 10.2196/15572

**Published:** 2020-06-09

**Authors:** Reham A Hameed Shalaby, Vincent I O Agyapong

**Affiliations:** 1 Department of Psychiatry University of Alberta Edmonton, AB Canada

**Keywords:** peer support, peer support workers, mental illness and addiction, social support, literature review

## Abstract

**Background:**

A growing gap has emerged between people with mental illness and health care professionals, which in recent years has been successfully closed through the adoption of peer support services (PSSs). Peer support in mental health has been variously defined in the literature and is simply known as the help and support that people with lived experience of mental illness or a learning disability can give to one another. Although PSSs date back to several centuries, it is only in the last few decades that these services have formally evolved, grown, and become an integral part of the health care system. Debates around peer support in mental health have been raised frequently in the literature. Although many authors have emphasized the utmost importance of incorporating peer support into the health care system to instill hope; to improve engagement, quality of life, self-confidence, and integrity; and to reduce the burden on the health care system, other studies suggest that there are neutral effects from integrating PSSs into health care systems, with a probable waste of resources.

**Objective:**

In this general review, we aimed to examine the literature, exploring the evolution, growth, types, function, generating tools, evaluation, challenges, and the effect of PSSs in the field of mental health and addiction. In addition, we aimed to describe PSSs in different, nonexhaustive contexts, as shown in the literature, that aims to draw attention to the proposed values of PSSs in such fields.

**Methods:**

The review was conducted through a general search of the literature on MEDLINE, Google Scholar, EMBASE, Scopus, Chemical Abstracts, and PsycINFO. Search terms included peer support, peer support in mental health, social support, peer, family support, and integrated care.

**Results:**

There is abundant literature defining and describing PSSs in different contexts as well as tracking their origins. Two main transformational concepts have been described, namely, intentional peer support and transformation from patients to peer support providers. The effects of PSSs are extensive and integrated into different fields, such as forensic PSSs, addiction, and mental health, and in different age groups and mental health condition severity. Satisfaction of and challenges to PSS integration have been clearly dependent on a number of factors and consequently impact the future prospect of this workforce.

**Conclusions:**

There is an internationally growing trend to adopt PSSs within addiction and mental health services, and despite the ongoing challenges, large sections of the current literature support the inclusion of peer support workers in the mental health care workforce. The feasibility and maintenance of a robust PSS in health care would only be possible through collaborative efforts and ongoing support and engagement from all health care practitioners, managers, and other stakeholders.

## Introduction

Peer support services (PSSs) are novel interventions recently adopted in mental health systems worldwide. It is believed, however, that PSSs date back to more than three centuries to the moral treatment era [1], albeit on an informal basis. Diverse definitions and classifications for PSSs have been provided in the literature [2-4], and numerous reports have praised and supported the service provided by peer support workers (PSWs) [5-8]. However, other literature suggests the neutral effects of PSSs, with weak associated evidence to support such services [9,10]. The potential impact of PSWs on their peers [11-14] has received considerable attention in the literature.

PSSs have been introduced in different contexts, such as family PSWs [15-19], the forensic field [20,21], and online PSSs. A considerable number of strategies were proposed to generate an effective PSS in the mental health field amid a number of associated concerns and challenges [22-25].

## Methods

This general review sheds light on PSWs’ experiences, benefits, challenges, opportunities to expand access to quality addiction, and mental health care using PSSs. The review was conducted through a general search of the literature on MEDLINE, Google Scholar, EMBASE, Scopus, Chemical Abstracts, and PsycINFO. Search terms included peer support, peer support in mental health, social support, peer, family support, and integrated care. We began the review with an examination of the definitions, origins, and types of peer support contributions and within different clinical contexts, aiming at deepening the view to the diverse effects of such a workforce. We then continued with examining the transition from a patient role to a PSW role and their incorporation into mental health systems. Thereafter, we provided a conceptual framework for the effects of peer support and stigma in relation to PSWs. We concluded the review by examining the benefits and challenges associated with PSSs and provided a commentary on future directions for PSSs in mental health.

## Results

### Definitions

Peer support has diverse meanings in the literature. For example, it is a *system of giving and receiving help founded on key principles of respect, shared responsibility, and an agreement of what is helpful* [26]. A *peer* is defined as *an equal*, someone with whom one shares demographic or social similarities, whereas *support* refers to “the kind of deeply felt empathy, encouragement, and assistance that people with shared experiences can offer one another within a reciprocal relationship” [3]. The Mental Health Foundation in the United Kingdom defined peer support in mental health as “the help and support that people with lived experience of a mental illness or a learning disability can give to one another” [27]. Peer employees were also defined as “individuals who fill designated unique peer positions as well as peers who are hired into traditional MH positions” [28]. In 1976, authors defined self-help groups as “voluntary small group structures for mutual aid in the accomplishment of a specific purpose...usually formed by peers who have come together for mutual assistance in satisfying a common need, overcoming a common handicap or life-disrupting problem, and bringing about desired social and/or personal change” [28]. Although the mutual relationship was sometimes overlooked and rather described as an asymmetric or nearly one-directional relationship [29], it is emphasized upon as 1 of the 4 main tasks for peer support accomplishments, which are mutuality, connection, worldwide, and moving toward rather than moving away [30].

### Origin and Growth of Peer Support

Davidson et al [11] have expressed the paradigm that calls for new models of community-based practice, which turned away from case management and from conceptualizing old practices under new terms. In the 1990s, peer support was formally introduced as a service in community mental health care. However, there is evidence of its practice throughout history, including during the moral treatment era in France at the end of the 18th century [1]. Recently, peer support has been rapidly growing in many countries and could attract a considerable amount of research [22]. Although Lunatic Friends’ Society is known as the earliest peer support group in mental health, which was founded in England in the middle of the 19th century [31], self-help groups were described as the oldest and most pervasive of peer support types [28]. Some peer-run groups also formed in Germany in the late 19th century, which protested on involuntary confinement laws. In addition to this, several individuals in the 18th and 19th centuries publicized their protests about their treatment in autobiographies and petitions [32]. The origin of peer support even reaches further back than the earliest asylums [33]. Some authors suggest that peer support is not based on psychiatric models and diagnostic criteria [3]; however, it is about “understanding another’s situation empathically through the shared experience of emotional and psychological pain” [34]. In the United States, the start of legitimacy for peer support was ignited in 2007 by considering the conditions under which PSSs could be reimbursed by Medicaid [35]. Although this reform was entailing a recovery model, which has been adopted by health care providers and stakeholders in many “English-speaking” countries, it was not the case in many other countries, in which this reform was yet to be well formulated [36].

### Transformational Concepts in Peer Support Service

#### Intentional Peer Support: Informal to Formal Peer Support Evolution

Intentional peer support (IPS) is described as a philosophical descendant of the informal peer support of the ex-patients’ movement in the 1970s [3]. It depends on a way of communication that immerses the provider into the recipient experience by stepping back from one’s story and being eagerly open to others’ stories [30]. In the field of psychiatry, trauma is blamed for playing a pivotal role in the experience, diagnosis, and treatment, and peer support is described as the logical environment for disseminating trauma-informed care (TIC) or service, which enables building relationships based on mutuality, shared power, and respect [37]. In the same context, trauma-informed peer support usually begins with the main question, “What happened to you?” instead of “What is wrong with you?” [30]. TIC is an explanatory model that identifies PSWs sharing lived experiences, ensuring safety and functioning as an advocate, and a liaison to patient management plans, where empowerment and intervention models are strongly emphasized upon [38,39]. The shift from a traditional biomedical model to recovery-oriented practice is meant to perceive trauma as a coping mechanism rather than a pathology [38,40]. This clearly entails training of all service providers for better acknowledgment and comfort in dealing with *trauma survivors*, with an understanding of trauma as an expectation rather than an exception [41]. Although the TIC concept has evolved over the years, it still lacks guidance, training, staff knowledge, and governmental support, which are necessary to ensure successful policy implementation [40]. The role of PSWs also extended to support those at risk of trauma events because of the nature of their work, including child protection workers, who are at risk of posttraumatic stress disorder or anxiety disorder [42]. Although IPS grew from the informal practices of grassroots-initiated peer support, it differs from earlier approaches because it is a theoretically based, manualized approach with clear goals and a fidelity tool for practitioners [14]. It instead focuses on the nature and purpose of the peer support relationship and its attention to skill building to purposefully engage in peer support relationships that promote mutual healing and growth [3]. Transitioning from informal to formal roles provides not only well-formulated expectations of the role but also a better chance to identify the potential conflict of the PSWs’ mixed identity [43].

Research conducted on PSWs has been conceptualized throughout history [22]. Starting with feasibility studies, at the initial stage, it is followed by studies comparing peer staff with nonpeer staff and, finally, the studies that answer questions such as the following:

- Do interventions provided by peers differ from those provided by nonpeers?
- What makes peer support a unique form of service delivery?

If so, to the previous question, what are the active ingredients of these aspects of peer support, and what outcomes can they produce?

Studies that provide answers to the latter set of questions are expected to provide a deeper understanding of the philosophical underpinnings of the IPS concept for PSSs.

#### The Transformation From Patient to Peer Support Providers

The shift from being a service recipient to a service provider has been contributing as a driving force to restore fundamental human rights, especially among those with serious mental illnesses (SMIs) [22]. Telling the personal lived experience leads to a profound shift, from telling an “illness story” to a “recovery story” [4]. This involved an identity transformation from being perceived as a victim or a patient to a person fully engaged in life with various opportunities ahead [4]. This transition is seen as a gradual process and one that is supported by several other personal changes with expected challenges [44]. Moving a full circle to include PSWs as the service provider has been undertaken by mental health services to further exceed the transformational role, which was primarily the main aim of providing such a service [45]. A liminal identity was given for PSWs as laying between several roles, being service users, friends, and staff. Thus, the professionalism of the PSW role might not be a successful way to ensure individual well-being or to promote the peer support initiative [46]. Thus, successful transitioning from the patient to PSW role involves fundamental functional shifts achieved through overcoming multiple barriers at the personal, health system, and societal levels.

### Effects of Peer Support Service in Different Contexts

Trained PSWs or mentors can use communication behaviors useful to different client groups. Many studies showed the effectiveness and feasibility of applying for peer support as follows:

#### Severe or Serious Mental Illness

Generally, the evidence for peer support interventions for people with SMIs has been described as moderate to limited with mixed intervention effects [2,47]. On the one hand, adding PSSs to intensive case management teams proved to improve *activation* in terms of knowledge, skills, confidence, and attitudes for managing health and treatment. Hence, patients become healthier, report better quality of life (QOL), engage in more health care practices, and report more treatment satisfaction [48,49]. On the other hand, a systematic review of randomized controlled trials (RCTs) involving adults with SMIs, while showing some evidence of positive effects on measures of hope, recovery, and empowerment at and beyond the end of the PSS intervention in this review, did not show any positive effects on hospitalization, satisfaction, or overall symptoms [10]. Similarly, a Cochrane systematic review of PSSs for people with schizophrenia found inconclusive results, with a high risk of bias in most of the studies and insufficient data to support or refute the PSS for this group [50].

#### Addiction and Drug Users

In recent years, peer recovery support services have become an accepted part of the treatment for substance use disorders, providing a more extensive array of services that are typically associated with the mutual supportive intervention [51]. This is in contrast to the use of peer support for SMIs where evidence is still developing. The Substance Abuse and Mental Health Services Administration (SAMHSA) defined peer recovery support for substance use disorders as “a set of nonclinical, peer-based activities that engage, educate, and support individuals so that they can make life changes that are necessary to recover from substance use disorders” [51]. Despite the long-term nature of substance abuse, immersion in peer support groups and activities and active engagement in the community are considered the 2 critical predictors of recovery for more than half the dependent substance users [52].

A number of trials studied the peer support effect on drug users, especially in the emergency department [53,54]. Another randomized trial found that a socially focused treatment can affect change in the patient’s social network and hence increase support for abstinence, for example, an increase of one nondrinking friend in the social network is translated into a 27% increase in the probability of reporting abstinence on 90% of days or more at all follow-up visits, which extended to 15 months [55].

#### Forensic Peer Support Service

The forensic peer system refers to the engagement of peer specialists who have histories of mental illness as well as criminal justice involvement and who are trained to help other patients sharing similar accounts [20]. As referred to by Davidson and Rowe [20], “Forensic Peer Specialists embody the potential for recovery for people who confront the dual stigmas associated with SMI and criminal justice system involvement.”

They offer day-to-day support for those released early from jail by accompanying them to initial probation meetings or treatment appointments and referring them to potential employers and landlords, helping people to negotiate and minimize continuing criminal sanctions and training professional staff on engaging consumers with criminal justice history [20,21]. PSWs with incarceration histories could successfully identify the liminal space in being supportive rather than providing support for the criminal offense, in contrast with the conventional methods that directly confront criminality [56]. In fact, having criminal history is the “critical component” for achieving recovery [56]. Multiple initiatives have been introduced to facilitate a reentry process for people recently released from incarceration, including Forensic Assertive Community Treatment, Assertive Community Treatment, Critical Time Intervention, and Women’s Initiative Supporting Health Transitions Clinic, through diverse community support groups involving PSWs [57,58].

#### Old Adults

A peer support program undertaken by older community volunteers was effective in improving general and physical health, social functioning, depression parameters, and social support satisfaction, especially in socially isolated, low-income older adults [59]. The Reclaiming Joy Peer Support intervention (a mental health intervention that pairs an older adult volunteer with a participant) has the potential for decreasing depression symptoms and improving QOL indicators for both anxiety and depression [60]. Engaging the community in health research could be of a high value in acknowledging their own health needs [61].

#### Youth and Adolescents

Peer support programs are mostly needed for university students, where challenges with loneliness and isolation are well recognized [62]. Hence, a need emerged for training peers to support their peer adolescents with the prospective challenges at this age [63]. Trained peer support students without necessarily having a lived experience were also examined in England [64]. The study included university students measuring the acceptability and impact of the volunteer peer support program through 6 weekly sessions. Students with lower mental well-being were more likely to complete the course, and an improvement in mental well-being was recorded for those who attended more frequently. Overall, peers remain to be an essential source of support for young people experiencing mental health and substance use problems [65].

#### Medically and Socially Disadvantaged Subgroups

A peer-led, medical self-management program intervention has been beneficial for medically and socially disadvantaged subgroups [60]. The Reclaiming Joy Peer Support intervention has the potential for increasing QOL and reducing depression in low-income older adults who have physical health conditions [60]. Similarly, for those who are “hardly reached,” it was indicated that the PSS provided is even more effective in these marginalized populations [66]. A Health and Recovery Peer program was delivered by mental health peer leaders for people with SMIs, resulting in an improvement in the physical health–related QOL parameters such as physical activity and medication adherence [49]. Peer-delivered and technology-supported interventions are feasible and acceptable and are associated with improvements in psychiatric, medical self-management skills, QOL, and empowerment of older adults with SMIs and or chronic health conditions [67,68].

#### Persons With Disabilities

The United Nations’ Convention on the Rights of Persons with Disabilities (CRPD) was adopted in 2007 and stated that “persons with disabilities should have equal recognition before the law and the right to exercise their legal capacity” [69-71]. Therefore, a positive emphasis upon the supported decision making and the fight against discrimination is evident through the convention. Nevertheless, these initiatives have been perceived as incomplete considering many challenges such as the community social status and ongoing perceived stigma of people with disabilities (PWDs) [70,72]. “Circle of support” is an elaborate example of an applicable peer support model for PWDs that has helped in decision making and facilitating communication [70,73,74]. This is clearly aligned with the paradigm shift from the biomedical to the socially supportive model of disability, which was provided by CRPD [70].

#### Peer Support for Families

Families may act either as sources of understanding and support or stigmatization through ignorance, prejudice, and discrimination, with subsequent negative impact [19]. In addition, the distress and burden associated with caring for a family member with mental illness are evident, where 29% to 60% endure significant psychological distress [17]. Family support can be financial or emotional; however, moral support was perceived as the substantial motivating factor for relatives who are ill [19]. In the last few decades, consistent and growing evidence that supports the inclusion of family members in the treatment and care of their misfortunate relatives has been developed. This has been mainly evident in the youth mental health system that urged the transformation change, which incorporates family members in the health care service provided to their youth [18,75]. Many PSWs have been engaged in family psychoeducation as family peers or parent partners, especially for those with the first episode of psychosis [76]. Although familial education is crucial and needs to be provided through different scales [19], an extensive matching of PSWs and the caregivers has not been perceived as a necessity to create a successful volunteer mentoring relationship [77]. Multiple initiatives have taken place all over the world. In India, a program titled “Saathi” was established for family members of residential and outpatient mental health service users that had dual goals of offering information and developing a peer support mechanism for family members of people with different mental health conditions [19]. In Melbourne, Australia, “Families Helping Families” was developed, where family PSWs are positioned in the service assessment area and in the *inpatient unit* to ensure early involvement [18]. An impressive peer support guide for parents of children or youth with mental health problems is provided by the Canadian Mental Health Association, British Colombia Division [15]. In Ontario, family matters programs are provided through provincial peer support programs [16].

The term “transforming mental health care” entails active involvement of families in orienting the mental health system toward recovery [78]. Family members are to have access to timely and accurate information that promotes learning, self-monitoring, and accountability [79]. The inclusion of family members as partners of the medical service is the new philosophy, with a subsequent shift from the concept of clinic-based practice to a community-based service approach [78].

#### Peer Support Service in Low- and Middle-Income Countries

Several initiatives took place in low- and middle-income countries, such as in rural Uganda, where a trained peer-led team provided 12 successful training sessions of perinatal service for a group of parents over a 6-month period, which resulted in better maternal well-being and child development, compared with another control group [80]. Similarly, successful community peer groups were conducted in rural India and Nepal, with high feasibility and effectiveness rates, and perceived as “potential alternative to health-worker-led interventions” [81-83]. In addition, adding counseling and social support groups entailing PSWs to the conventional medication treatment for patients with psychotic disorders was tried in a cohort study in Uganda; however, the results were not significantly different from those who received only medications [84]. This might be because of the underpowering of community services offered, compared with the robust medication regimens [85].

It is evident from the aforementioned information that there is mixed evidence on the effectiveness of PSW interventions in different contexts. For example, for patients with SMIs, systematic reviews suggest that there is some evidence of positive effects on measures of hope, recovery, and empowerment but no positive effects on hospitalization, satisfaction, or overall symptoms [10]. Similarly, for patients with addiction issues, although being involved in a peer network did not reduce social assistance for alcohol, they somewhat increased behavioral and attitudinal support for abstinence as well as involvement with Alcoholics Anonymous [55]. Furthermore, although many observational studies support the PSW role in the other contexts described above, there is a current dearth of literature involving RCTs and systematic reviews reporting on the effectiveness of PSWs in these specific contexts. Thus, there exist opportunities for conducting RCTs in the described contexts.

### The Conceptual Framework for the Effects of Peer Support Service

**Figure 1:**
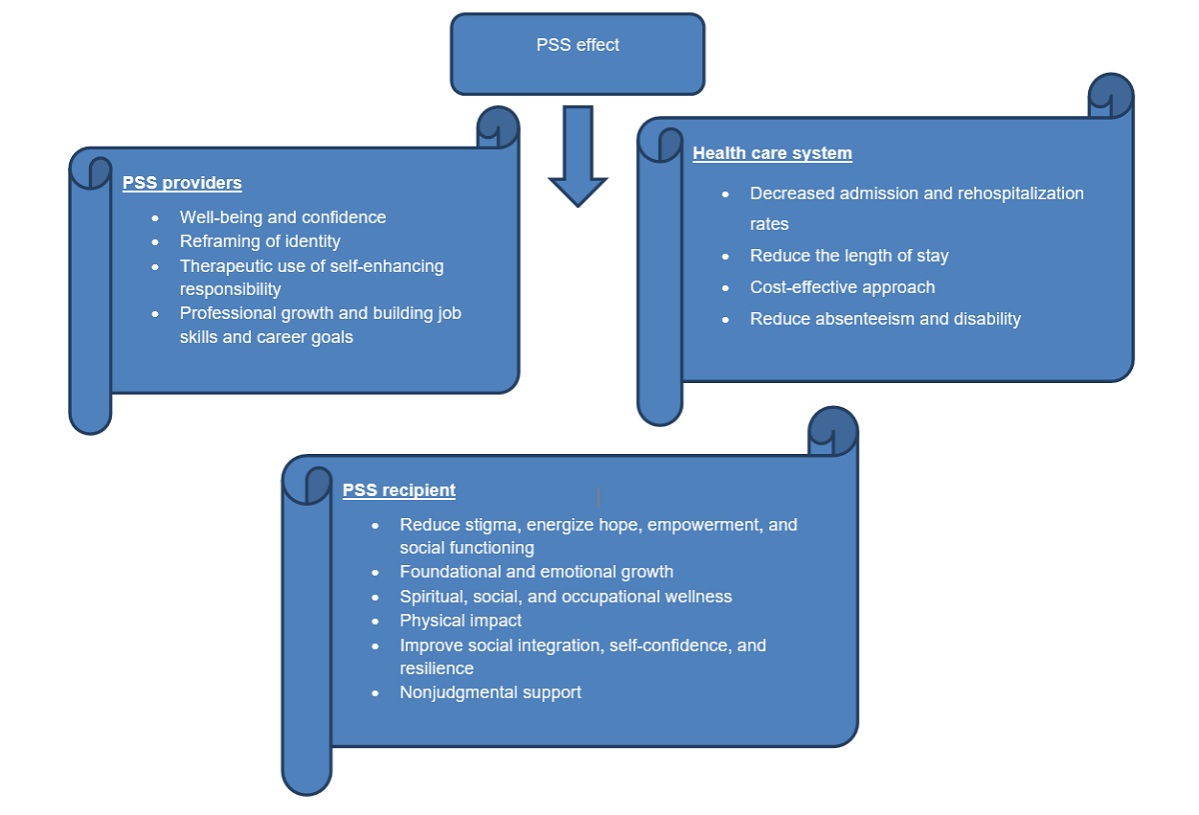
The conceptual framework for the impact of peer support workers in mental health. MH: mental health; PSS: peer support service; PSW: peer support worker.

The conceptual framework is based on empirical evidence, suggesting that the impact of PSWs reflects upon the recipients of such a service [4,76,86-90], the global health system [22,47,76,86,91,92], and the PSWs themselves [13,28,76,93], as shown in Figure 1. The framework has, therefore, been developed by authors through a general review of the literature that examines the effects of PSSs on patients, health care systems, and also PSWs themselves so as to provide evidence-based material supporting all possible effects of PSW roles.

Supportive social relationships can have a dual opposing effect on individuals’ lives, either as a family member or as social and professional networks through sharing their disappointments and pains or their joy and successes [11]. Useful roles for PSSs are identified in many studies. For example, adding 3 peer specialists to a team of 10 intensive case managers provided better QOL with greater satisfaction [12], stigma reduction, and less health service utilization [89,91]. The economic impact of PSSs has been extensively studied in the literature, concluding cost containment for the health care system in terms of reduction of readmission rates, emergency visits, and fewer hospital stays, which altogether substantially exceed the cost of running a peer support program [92]. Moreover, PSWs are looked at as providers of a service at a cheaper cost compared with other health care providers [94,95]. For example, about US $23,000 is paid to PSWs in the United States compared with around US $100,000 for a nurse practitioner [96]. However, a PSS is not posited as a substitute for clinical services, rather it is perceived as an intrapersonal and social service that provides a dual role of effective service and with humanizing care and support [14,26,97]. This role extends to cover PSWs themselves, in terms of improved overall well-being and self-confidence, reframing identity, and enhancing responsibility either toward themselves or their peers [13,93].

Although PSWs can play a variety of tasks, managers who hire them may want to ensure that improving patient activation is included in their range of duties [48]. In 2 concurrent studies, a significant increase in QOL satisfaction, reduction of rehospitalization rates, and reduction in the number of hospital days were recorded when adding PSSs to usual care [22,98]. In another study engaging 31 peer providers in diverse mental health, agencies identified 5 broad domains of wellness, including foundational, emotional, growth and spiritual, social, and occupational wellness [4]. In a systematic literature review for people with SMIs, peer-navigator interventions and self-management were the most promising interventions [47]. PSWs’ effects are diversified through sharing in different contexts. For example, positive impacts on the physical health of their peers have been recorded [49]. Peer-based approaches have been used to deliver behavioral weight loss interventions [90]. For young students, structured peer support for depression may have benefits in improving students’ mental well-being [64]. In the case of crisis houses, greater satisfaction was achieved through a provided informal PSS [99]. Robust studies, therefore, recommend implementing peer support programs [10,18].

On the other hand, authors found that PSSs met moderate levels of evidence and that effectiveness varied across service types, for example, with “peers in existing clinical roles” was described as being less effective than the “peer staff added to traditional services” and “peer staff delivering structured curricula” [3]. Other reviews suggested that current evidence does not support recommendations or mandatory requirements from policy makers to offer programs for peer support [9,10].

### Peer Support Workers’ Satisfaction and Challenges

PSWs experience different problems alongside their diverse job roles, including low pay, stigma, unclear work roles, alienation, struggling with skill deficits, lack of training opportunities, emotional stress in helping others, and, on top of that, maintaining their personal physical and mental health wellness [100,101]. Researchers found that PSWs experience discrimination and prejudice from nonpeer workers, in addition to the encountered difficulties of how to manage the transition from being a patient to a PSW. As a result, high attrition rates were noted among PSWs in mental health settings [102,103]. Peer job satisfaction is strongly dependent on several factors [100,104,105]. Role clarity and psychological empowerment, organizational culture, and working partnership with peers were the most significant predictors of PSW job satisfaction, while professional prejudice was not perceived as a significant predictor [106,107]. Other studies noted that the main problems were experiencing marginalization, lack of understanding, and a sense of exclusion [108-110]. Payment could also contribute to the amount of satisfaction of PSWs [76], as compensation helps through facilitation and engagement motivation [109]. Nevertheless, it seems that not the payment, which ranged from US $10 to US $20 per hour, but the lack of recognition and acknowledgment are the causes for job nonsatisfaction [104].

An interesting literature review grouped these challenges and barriers facing PSWs during fulfilling their assigned roles into 6 main categories: nature of the innovation, individual professional, service user, social context, organizational context, and economic and political contexts [111].

It is evident from the abovementioned information that the PSW role is challenged at multiple levels, including at the personal, societal, and organizational levels. These challenges have a direct bearing on PSW satisfaction, and the successful integration of the PSW role into the health care system depends to a great extent on how these challenges are overcome.

### Novel Technology in Peer Support Service (Online and Telephone)

Online support groups are usually conducted through bulletin boards, emails, or live chatting software [28]. Online groups are familiar with people whose illnesses are similar to SMIs or affecting the body shape that have forced them to experience embarrassment and social stigmatization [23,24]. Therefore, they split from the social contexts and redirect toward novel ways of help, such as PSWs and online support groups, and web-based communities provided a suitable medium for people with SMIs by following and learning from their peers on the web, which positively helped them to fight against stigma, instilling hope and gaining insight and empowerment for better health control [25]. Increasingly, social media grew as a target for individuals with SMIs, such as schizophrenia, schizoaffective disorder, or bipolar disorder, seeking advice and supporting each other [112-114]. For someone with SMIs, the decision to reach out and connect with others typically occurs at a time of increased instability or when facing significant life challenges [115]. In a qualitative study, popular social media, such as YouTube, appeared useful for allowing people with SMIs to feel less alone, find hope, support each other, and share personal experiences and coping strategies with day-to-day challenges of living with mental illness through listening and posting comments [114]. Mobile phone–based peer support was found to be a feasible and acceptable way to the youngsters during their pregnancy as well as in the postpartum period [116]. In addition, when coupled with frequent face-to-face meetings with PSWs and with “text for support,” it could be of high value for patients with different mental illnesses [117]. Although online peer networks actively fight against discrimination and stigma, their accessibility to diverse patients’ sectors regarding their income and ethnicity is still questionable [25].

### Future of Peer Support Services

Potential new roles, such as community health workers, peer whole health coaches, peer wellness coaches, and peer navigators, have been suggested for such a workforce [76]. They are described as an “ill-defined potential new layer of professionals” [118]. Through an initiative undertaken by SAMHSA via its “Bringing Recovery Supports to Scale Technical Assistance Center Strategy,” a successful identification of abilities and critical knowledge necessarily required for PSWs who provide help and support for those recovering from mental health and substance abuse was noted [76]. At present, peer support is seen as a growing paradigm in many countries, including the United Kingdom, Canada, New Zealand, France, and the Netherlands [103,119]. As an evolving culture, peer support has the opportunity to forge not just mental health system change but social change as well [37]. A novel peer support system termed “Edmonton peer support system” (EPSS) is currently being tested in a randomized controlled pilot trial [117]. In this study, investigators are evaluating the effectiveness of an innovative peer support program that incorporates leadership training, mentorship, recognition, and reward systems for PSWs, coupled with automated daily supportive text messaging, which has proven effectiveness in feasibility trials involving patients with depression and alcohol use disorders [120,121]. Previous studies have examined the effect of PSSs in different contexts, including outpatient departments [122], emergency departments [53,54], community mental health clinics [123,124], and inpatient sites [125]. On the contrary, the EPSS study focuses on patients who have been discharged from acute care hospitals. These patients are being randomized into 1 of the 4 main groups: enrollment in a peer support system, enrollment in a peer support system plus automated daily supportive and reminder text messages, enrollment in automated daily supportive and reminder text messages alone, or treatment as usual follow-up care. The research team hypothesizes that patients who are assigned to a peer support system plus automated daily supportive and reminder text messages will show the best outcome.

Organizations may facilitate peer support through their values, actions, and oversight [119] and through a robust supervision system with available educational access, which could be the adequate path for creating a positive and risk-free environment for PSWs throughout their complex workloads [126]. On the other hand, ethics committees play essential roles in the inclusion of PSWs in applied research studies by avoiding repetition of the work of other trusted agencies and considering the ethical validity of consent procedures for peer support interventions [127].

## Discussion

There is an internationally growing trend to adopt PSSs within addiction and mental health services, and despite the ongoing challenges, large sections of the current literature support the inclusion of the PSWs into the mental health care workforce. The literature suggests that the benefits of PSSs impact not only the recipients of mental health services but also extend to the PSWs and the whole health care system. Although the expected benefits of PSSs might be directly measured in terms of service utilization or patient improvement indicators, this could also extend to include wellness and empowerment for PSWs, who may still be fragile, vulnerable, and in need of ongoing acknowledgment and recognition. Thus, the potential for PSSs to be embedded into routine care and the opportunities for the development of innovative models of care for addiction and mental health patients such as the EPSS, which incorporates PSSs and supportive text messaging [117], are evidently a high valued priority. However, the feasibility and maintenance of a robust PSS in health care would only be possible through collaborative efforts and ongoing support and engagement from all health care practitioners, managers, and other stakeholders.

This literature review has several limitations. First, the review is not a systematic review or meta-analysis, and as such, there were no well-defined inclusion or exclusion criteria of studies, which potentially could lead to the exclusion of some essential related studies. Second, the search was conducted in English publications only. Consequently, there is a high probability of missing critical related publications published in non-English languages. Finally, as the review depended mainly on the available literature from the aforementioned sources, which showed marked variability in their design and covered diverse ideas under the central theme, the different weights for each idea throughout the review could be noted.

## Abbreviations

CRPD: Convention on the Rights of Persons with Disabilities
EPSS: Edmonton peer support system
IPS: intentional peer support
PSS: peer support service
PSW: peer support worker
PWD: people with disability
QOL: quality of life
RCT: randomized controlled trial
SAMHSA: Substance Abuse and Mental Health Services Administration
SMI: serious mental illness
TIC: trauma-informed care
